# Identification and Functional Analysis of Three *NlCstF* Genes in *Nilaparvata lugens*

**DOI:** 10.3390/insects15110867

**Published:** 2024-11-05

**Authors:** Shengli Jing, Feifei Wang, Aobo Ren, Fang Zheng, Bingbing Yu, Jingang Xu, Yali Liu, Jing Yang, Ruixian Chen, Wei Zeng, Yimei Zhang, Danxia Ke, Xiantao Ma, Hengmin Tang, Qingsong Liu, Bin Yu

**Affiliations:** 1 College of Life Sciences, Xinyang Normal University, Xinyang 464000, China; ffwang202203@163.com (F.W.); renaobo202202@163.com (A.R.); zf15837634522@163.com (F.Z.); yubingbing2021@163.com (B.Y.); xjg157164@163.com (J.X.); liuyali_020601@163.com (Y.L.); jingyang2024@163.com (J.Y.); chenruixian2002@163.com (R.C.); zw@xynu.edu.cn (W.Z.); xynuym@163.com (Y.Z.); kdx_029@163.com (D.K.); thm1582718@xynu.edu.cn (H.T.); 2 College of Chemistry and Chemical Engineering, Xinyang Normal University, Xinyang 464000, China; xiantaoma@xynu.edu.cn; 3 State Key Laboratory of Cotton Bio-Breeding and Integrated Utilization, State Key Laboratory of Crop Stress Adaptation and Improvement, Key Laboratory of Plant Stress Biology, School of Life Sciences, Henan University, Kaifeng 475004, China

**Keywords:** brown planthopper, *NlCstF* genes, lethal phenotypes, reproduction, molting disruption

## Abstract

The Cleavage Stimulation Factor (CstF) complex, consisting of three subunits, is essential for the 3′ end processing of precursor messenger RNA (pre-mRNA). In mammals, this complex includes CstF50, CstF64, and CstF77, named according to their molecular weights, and these proteins are conserved across many organisms. However, the functional roles of the three CstF genes (*NlCstF50*, *NlCstF64*, and *NlCstF77*) in *Nilaparvata lugens*, a major rice pest, have not been fully explored. This study identified and characterized the sequences of these genes, with proteins encoded by *NlCstF50*, *NlCstF64*, and *NlCstF77* consisting of 439, 419, and 732 amino acids, respectively. These proteins are conserved among various insect species. Spatio-temporal expression analysis revealed that these genes are expressed at all developmental stages and in various tissues, with peak levels in eggs and testes. RNA interference (RNAi) targeting one or all three *NlCstF* genes resulted in a reduction in gene expression by 68% to 90% at 72 h post-injection, indicating that multi-gene dsRNA can achieve similar silencing outcomes as single-gene dsRNA. Knocking down one or all three *NlCstF* genes caused significant lethal phenotypes and molting disruptions. Mortality rates increased from 62.5% (ds*NlCstF50*) to 95.4% (ds*NlCstF(50+64+77)*). Additionally, silencing these genes reduced the number of eggs laid per female and hatch rates. These results highlight the critical role of *NlCstF* genes in the development and reproduction of *N. lugens*, suggesting their potential as targets for RNAi-based pest control strategies.

## Simple Summary

The brown planthopper is a major pest threatening rice crops in Asia, making it essential to identify target genes for RNAi-based pest control strategies. The Cleavage Stimulation Factor (CstF) complex, which plays a key role in mRNA 3′ end processing, is composed of several critical genes, and mutations in these genes can cause severe developmental defects, indicating their potential as targets. In this study, we identified homologs of the human CstF complex genes (*NlCstF50*, *NlCstF64*, and *NlCstF77*) in *Nilaparvata lugens* and investigated their functions using RNAi techniques. These genes are expressed throughout all developmental stages and various tissues, with particularly high levels in eggs and testes. Targeting these genes with RNAi resulted in significant mortality, decreased egg production, and lower hatch rates. These results suggest that *NlCstF50*, *NlCstF64*, and *NlCstF77* could be effective targets for RNAi-based pest control strategies.

## 1. Introduction

In eukaryotes, the formation of the 3′ end of almost all messenger RNA (mRNA) is crucial for transcription termination and mRNA export [1]. This 3′ end processing mechanism involves two steps: cleavage and polyadenylation, which require the interaction of pre-mRNA cis elements with more than 14 proteins [2]. These proteins form various complex factors that recognize the cis element sequences. In mammals, there are five major factors: Cleavage and Polyadenylation Specificity Factor (CPSF), Cleavage Stimulatory Factor (CstF), Cleavage Factors I (CFI) and II (CFII), and poly(A) polymerase (PAP) [3,4,5].

CstF is a highly conserved protein complex composed of three subunits that bind to U- and GU-rich sequences downstream of the polyadenylation signal of precursor mRNAs (pre-mRNAs) [6,7,8]. In humans, these three subunits are termed CstF50, CstF64, and CstF77, corresponding to their molecular weights [6]. CstF50 contains an N-terminal dimerization domain that can self-associate [9], and seven beta-transducin (WD40) repeats that interact with CstF77 [10,11] and with the carboxy-terminal domain (CTD) of the largest subunit of RNA polymerase II (Pol II) [12]. CstF50 is found exclusively in multicellular eukaryotes and lacks any known homolog in yeast [8]. CstF64 possesses an N-terminal RNA-binding domain, known as an RNA-recognition motif (RRM) [13]. Functional and structural studies have shown that RRM recognizes U- and GU-rich sequences of pre-mRNAs and directs the cleavage site [7,14]. The region following the RRM, known as the hinge region, interacts with CstF77 but not with CstF50 [11,15]. The C-terminal domain (CTD) of CstF64 is highly conserved across all eukaryotes [16]. In yeast, the absence of the CTD in Rna15, the yeast counterpart of CstF64, leads to slow growth or cell death in vivo [16].

CstF77 contains twelve HAT (half-a-TPR) domains at the N-terminus [17]. Functionally, these repeated domains are required for intrinsic dimerization [18,19]. Another important region in the C-terminal CstF77 is the proline-rich segment, which is critical for protein–protein interactions. This segment interacts with the hinge region of CstF64 and with the WD40 repeats of CstF50 [11]. Thus, CstF77 is central in the hexameric CstF complex, acting as a scaffold to bridge both CstF64 and CstF50 [19]. CstF77 is highly conserved and has homologs in all eukaryotes, such as *Drosophila* Su(f) (Suppressor of forked) [20,21], yeast RNA14 [22], and *Arabidopsis* AtCstF77 [23]. Mutation of the Su(f) gene in *Drosophila* causes a temperature-sensitive phenotype of lethality and female sterility [24] and results in defects in cell proliferation [25]. Similar phenotypes of lethality and loss of cell viability are observed in RNA14 and RNA15 mutants in yeast [26]. Mutation of AtCstF77 in *Arabidopsis* causes severe developmental defects [27,28]. These phenotypes are associated with the function of CstF77, suggesting that the CstF complex plays a crucial role in mRNA 3′ end processing. However, the function of CstF complex genes in Hemiptera insects has not been studied.

The brown planthopper (BPH, *Nilaparvata lugens*) has been a significant pest threatening rice production in Asia for several decades [29]. With advancements in genome and transcriptome sequencing, along with powerful gene function tools like RNA interference (RNAi), BPH has become the model insect for the Hemiptera order. Despite these advancements, the function of the CstF complex genes in *N. lugens* remains unclear. In this study, based on orthology to humans, the three CstF complex genes of BPH (*NlCstF50*, *NlCstF64*, and *NlCstF77*) were cloned, and their phylogenetic relationships and spatiotemporal expression patterns were analyzed. RNA interference (RNAi) was used to investigate the biological functions of *NlCstF50*, *NlCstF64*, and *NlCstF77*. Knockdown of one or all of these three genes caused significant lethality in BPH, along with a reduction in the number of eggs laid by females and the hatchability of both females and males. These results indicate that *CstF* genes play a critical role in the development and fecundity of this insect.

## 2. Materials and Methods

### 2.1. Insects

The *N. lugens* specimens were initially obtained from Wuhan University, China. For this study, the TN1 (Taichuang Native 1) variety of rice seedlings was used to feed the BPH. The experimental BPH insects have been maintained by our research group in an artificial climate room for over nine years. The rearing conditions were as follows: a temperature of 26 ± 2 °C, a photoperiod of 16 h light and 8 h dark, and a relative humidity of approximately 65 ± 5%.

### 2.2. Total RNA Extraction, cDNA Synthesis and Cloning of Three NlCstF Genes

Total RNA was extracted from BPH across various developmental stages and tissues using the RNAiso Plus Kit (Takara, Dalian, China). Following the manufacturer’s protocol, each sample was lysed in 100 µL TRIzol, deproteinized with chloroform, precipitated with isopropanol, and washed with 75% ethanol. RNA concentration was determined using a Nanodrop 2000 spectrophotometer (Thermo Fisher Scientific, Waltham, MA, USA). First-strand cDNA was synthesized from 1 µg of total RNA using the PrimeScript™ RT Reagent Kit with gDNA Eraser (Takara, Dalian, China), following the reagent kit instructions.

For cloning the three *NlCstF* genes, cDNAs were used as templates. Primers were designed using Primer Premier 5.0 software, as detailed in Table S1. The coding sequences (CDS) for these genes were obtained from the National Center for Biotechnology Information (NCBI) database (http://www.ncbi.nlm.nih.gov/) (accessed on 2 August 2024) with the following GenBank accession numbers: XM_039444747 for *NlCstF50*; XM_039439518 for *NlCstF64*; XM_02234360 for *NlCstF77*. Due to the challenges in amplifying these genes, 2–3 pairs of specific primers (Table S1) were designed for each gene to ensure successful amplification. The 50 µL PCR reaction mixture included 0.25 µL Ex-Taq DNA Polymerase, 5 µL 10× Ex Taq Buffer, 4 µL 2.5 mM dNTP, 1 µL of each forward and reverse primer, 1 µL cDNA template, and 37.75 µL ddH_2_O. The PCR amplification protocol consisted of an initial denaturation at 95 °C for 5 min, followed by 35 cycles of denaturation at 95 °C for 30 s, annealing at approximately 60 °C for 30 s, and extension at 72 °C for 1.5 min, with a final extension at 72 °C for 10 min. The quality of the PCR products was assessed via agarose gel electrophoresis. The PCR products were then ligated into the pMD18-T vector (Takara, Dalian, China), transformed into *Escherichia coli* DH5 α competent cells, and positive clones confirmed by agarose gel electrophoresis were sent for sequencing by Wuhan HeTaiQing Biological Company.

### 2.3. Sequence and Phylogenetic Analysis

Homologous proteins for the three *Nl*CstF proteins were identified using the BLAST program 2.13.0 in the NCBI database (https://blast.ncbi.nlm.nih.gov) (accessed on 9 August 2024). The amino acid sequences of these homologous proteins were aligned using Jalview 2.11.3.3 software. Conserved domains within the three *NlCstF* genes were predicted using the NCBI Conserved Domain Search tool 2.16 (https://www.ncbi.nlm.nih.gov/Structure/cdd/wrpsb.cgi) (accessed on 9 August 2024) in conjunction with relevant literature. A phylogenetic tree was constructed using the Neighbor-Joining method in MEGA 6 software and bootstrapped with 1000 replications [30], the values being expressed as percentages. The accession numbers of the sequences used for multiple sequence alignment and phylogenetic analysis are provided in Table S2.

### 2.4. Expression Pattern Analysis of NlCstFs and qRT-PCR

To investigate the developmental and tissue-specific expression patterns of the three *NlCstF* genes, samples were obtained from BPH at various developmental stages, including eggs (n = 100), 1st- to 5th-instar nymphs (n = 50–80), and adult females and males (n = 10). Additionally, samples from different tissues, such as salivary glands (n = 20), guts (n = 15), fat bodies (n = 20), ovaries (n = 10), and testes (n = 10), were collected from adult females and males 72 h post-emergence. All samples were collected in triplicate for biological replication.

Total RNA was extracted and reverse-transcribed from the various developmental stages and tissues using the previously described method, to facilitate quantitative real-time PCR (qRT-PCR) analysis of the three *NlCstF* genes. Gene-specific primers for qRT-PCR were designed using Primer Premier 5 software (Table S1), based on the sequences of the cloned *NlCstF* genes.

The qRT-PCR reactions were carried out in a total volume of 10 µL, comprising 5 µL of 2 × M5 HiPer SYBR Premix Es Taq (Mei5bio, Beijing, China), 2.6 µL of H_2_O, 0.4 µL of each primer, and 2 µL of 10-fold diluted cDNA. The qRT-PCR was performed on a CFX96TM real-time PCR detection system (Bio-Rad, Philadelphia, PA, USA) with the following parameters: initial denaturation at 95 °C for 60 s, followed by 40 cycles of 95 °C for 10 s and 60 °C for 60 s.

The relative expression levels of the three *NlCstF* genes across various samples were quantified using the *18S ribosomal RNA* gene (*Nl18S rRNA*, GenBank accession number: JN662398) as the internal reference. Each sample was analyzed in at least three biological replicates. The final relative expression levels of the *NlCstF* genes were calculated using the 2^−ΔΔCt^ (Ct: cycle threshold) method [31].

### 2.5. Double-Stranded RNA (dsRNA) Synthesis and RNAi

Based on the different purpose of the RNAi experiments, the different dsRNAs were injected into third- and fifth-instar nymphs, respectively. The nymphs were anaesthetized with carbon dioxide for 15–20 s, and 23 nL of dsRNA (1 μg/μL) was microinjected into the mesothorax of each insect using a microprocessor-controlled Nanoliter 2020 injector (World Precision Instrument, Sarasota, FL, USA) [32]. After injection, the nymphs were transferred to fresh TN1 rice seedlings. At 72 h post-injection, total RNA was extracted from four randomly selected nymphs from each group for qRT-PCR analysis, adhering to the protocols previously described. To ensure data accuracy, each group sample was analyzed in three biological replicates.

To target the *NlCstF* genes and the green fluorescent protein gene (*GFP*) from *Aequorea victoria* for RNA interference (RNAi) studies, dsRNA-specific primers containing the T7 promoter sequence were designed based on the sequences of *NlCstF50*, *NlCstF64, NlCstF77,* and *GFP* (Table S1). Plasmids containing the correct sequences of the three *NlCstF* genes and *GFP* served as templates for PCR amplification using dsRNA-specific primers. The PCR products were then purified and used for dsRNA synthesis, following the manufacturer’s instructions for the MEGA script T7 High Yield Transcription Kit (Ambion, Austin, TX, USA). The quality and concentration of gene-specific dsRNAs (ds*NlCstF50*, ds*NlCstF64*, ds*NlCstF77*, and ds*GFP*) were determined by 1% agarose gel electrophoresis and NanoDrop 2000 spectrophotometry (Thermo Fisher Scientific, Waltham, MA, USA) [32]. The final dsRNA concentration was adjusted to 1 μg/μL using DEPC-treated water. To simultaneously silence the expression of the three *NlCstF* genes, a mixture of dsRNAs (ds*NlCstF(50+64+77)*) was prepared by combining equal amounts of ds*NlCstF50,* ds*NlCstF64*, and ds*NlCstF77*, maintaining a concentration of 1 μg/μL, similar to that of the single gene dsRNAs.

### 2.6. Effect of RNAi on the Survival of BPH

To investigate the effects of RNAi targeting *NlCstF* genes on the survival of BPH, 50–60 third-instar nymphs per biological replicate were injected with dsRNAs and subsequently reared on 35-day-old TN1 rice seedlings. Each group of nymphs, injected with different dsRNAs, was analyzed in at least three biological replicates. The number of surviving insects was recorded daily for 12 days post-injection. The survival rate was calculated as the ratio of the number of live insects to the initial number of insects.

To observe the lethal phenotypes in BPH, morphological changes in third-instar nymphs injected with dsRNAs were examined under a stereomicroscope (Leica S8APO, Wetzlar, Germany). Based on these observations, the lethal phenotypes were categorized primarily into two types associated with molting disruption. One, the old cuticle splits open at the notum, exposing the underlying notum (notum split). Two, the old cuticle remains attached to the abdomen and hind legs of the BPH, resulting in unsuccessful shedding during the eclosion period (deficient molting). The number of insects exhibiting each phenotype category was meticulously recorded. The percentage of each phenotype category was calculated by dividing the number of insects in each category by the initial number of insects.

### 2.7. Effect of RNAi on Reproduction of BPH

To investigate the effect of RNAi on the reproduction of BPH, a sufficient number of adult insects are required for mating experiments. However, injecting third-instar nymphs with dsRNAs targeting the *NlCstF* genes resulted in high mortality rates, thus reducing the number of adult insects available for further reproductive studies. Therefore, fifth-instar nymphs were selected for the injection of four different dsRNAs targeting the *NlCstF* genes.

Newly emerged female and male adults were separately reared on fresh rice seedlings in different containers for a period of three days. Subsequently, one female injected with a specific dsRNA was mated with one male injected with either the same or a different dsRNA on a single rice seedling enclosed in a casing device (height = 80 mm). After copulation, the females laid eggs on the leaf sheaths of the rice seedlings for three days. Both female and male adults were then removed and stored at −80 °C for further analysis. Four days later, the number of newly hatched nymphs from each mating pair was recorded daily for 8–10 consecutive days. The recording was terminated when no more hatching nymphs were observed. Unhatched eggs in the rice leaf sheaths were dissected and counted under a stereomicroscope (Leica S8APO, Wetzlar, Germany). The total number of eggs laid per female was calculated by summing the hatched nymphs and unhatched eggs. The hatching rate was determined by dividing the number of hatched nymphs by the total number of eggs laid per female.

Based on the combinations of mating females and males, there were three types of mating pairs. Firstly, both female and male were injected with the same type of dsRNA. Secondly, females injected with ds*GFP* were mated with males injected with any of the five different dsRNAs. Thirdly, males injected with ds*GFP* were mated with females injected with any of the five different dsRNAs.

To further understand the RNAi effect on reproduction, the morphology and internal reproductive organs of three-day post-emergence adults injected with different dsRNAs were observed under a stereomicroscope (Leica S8APO, Wetzlar, Germany). To ensure successful mating, females that had completed egg-laying three days post-copulation were dissected. Successful copulation was confirmed by the presence of spermatophores within the bursa copulatrix of these females.

### 2.8. Data Analysis

Statistical analyses were conducted using SPSS 22.0 software (IBM, New York, NY, USA). Data were presented as means ± standard error of the mean (SEM). Statistical significance was assessed using Student’s *t*-test (* *p* < 0.05; ** *p* < 0.01; *** *p* < 0.001) or one-way ANOVA. Images were captured with a stereomicroscope (Leica S8APO, Wetzlar, Germany).

## 3. Results

### 3.1. Sequence and Phylogenetic Analysis of Three NlCstF Genes

To elucidate the potential functions of CstF proteins, we cloned and sequenced the full coding sequences of three CstF genes in *N. lugens*: *NlCstF50*, *NlCstF64*, and *NlCstF77*, encoding proteins of 439, 419, and 732 amino acids, respectively.

**Figure 1:**
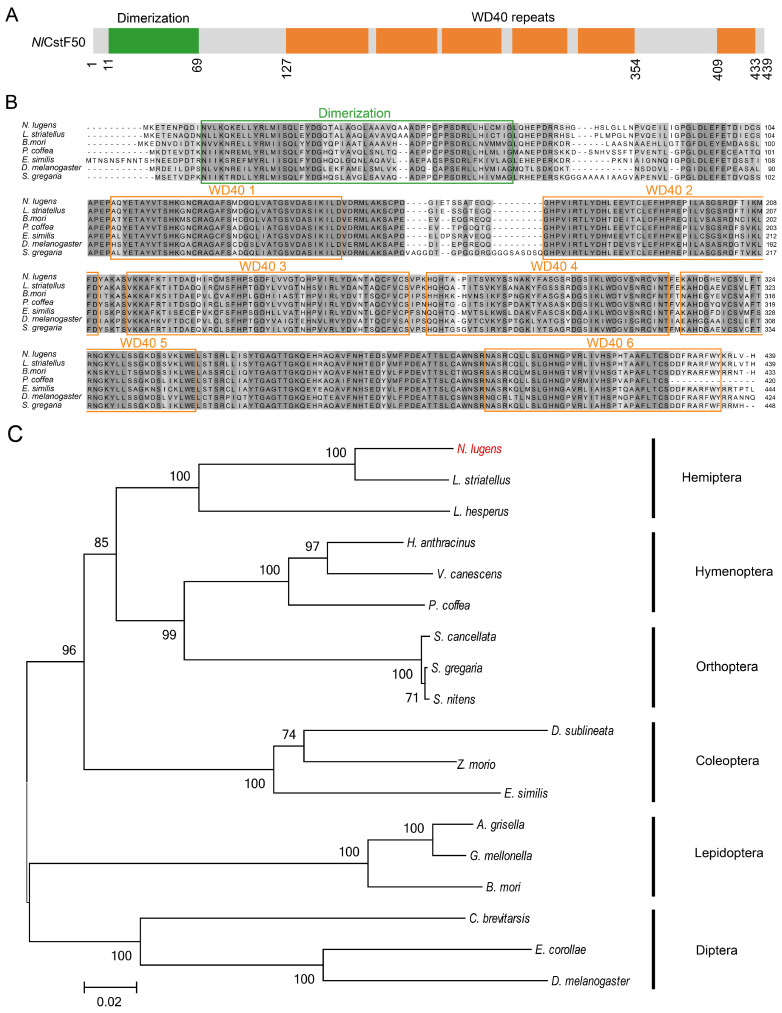
Sequence and phylogenetic analysis of *Nl*CstF50. (**A**) Schematic representation of the domain organization in the *Nl*CstF50 protein. (**B**) Amino acid sequences alignment across six insect orders: *Laodelphax striatellus* (Hemiptera), *Bombyx mori* (Lepidoptera), *Phymastichus coffea* (Hymenoptera), *Euwallacea similis* (Coleoptera), *Drosophila melanogaster* (Diptera), and *Schistocerca gregaria* (Orthoptera). The green and orange boxes indicate the dimerization and the WD40 repeat domains, respectively. (**C**) A phylogenetic tree was constructed using the maximum likelihood method in MEGA6, based on *Nl*CstF50 and 17 orthologs from six different insect orders.

Firstly, *Nl*CstF50 contains an N-terminal dimerization domain and six WD40 repeats at its C-terminus (Figure 1A). We retrieved six orthologs of CstF50 from six different insect orders using the NCBI database for alignment analysis. The alignment of *Nl*CstF50 with predicted amino acid sequences showed highly conserved dimerization and WD40 domains across insect species (Figure 1B). Phylogenetic tree analysis of seventeen CstF50 orthologs indicated that *Nl*CstF50 is conserved across various insect orders (Figure 1C).

**Figure 2:**
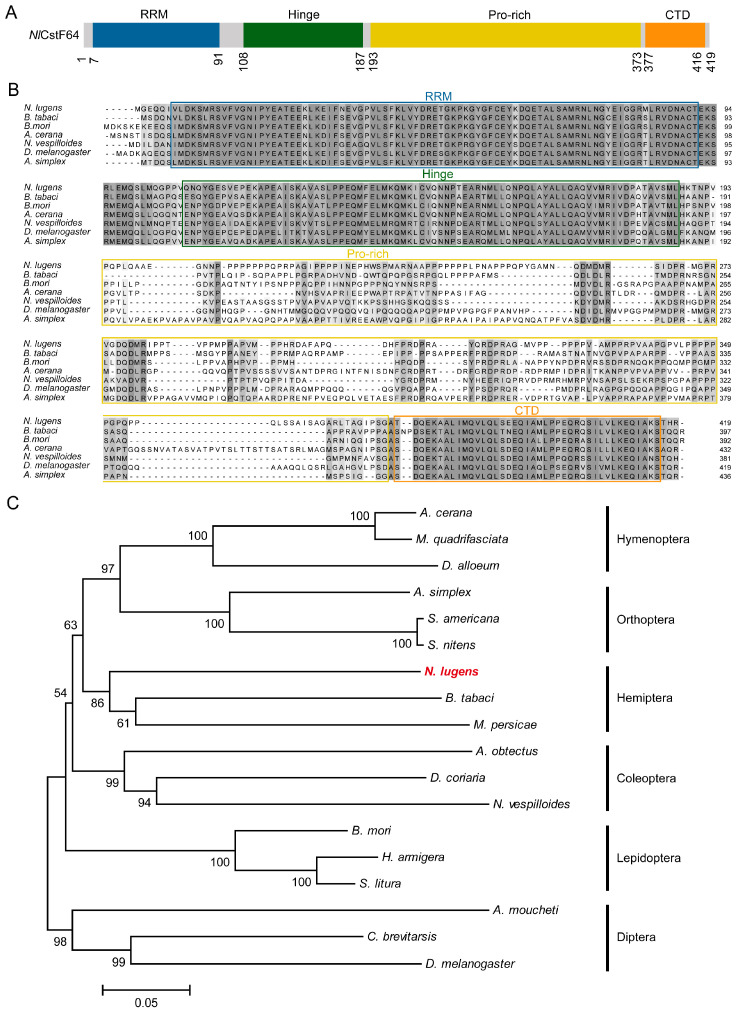
Sequence and phylogenetic analysis of *Nl*CstF64. (**A**) Schematic representation of the domain organization in the *Nl*CstF64 protein. (**B**) Alignment of amino acid sequences across six insect orders: *Bemisia tabaci* (Hemiptera), *Bombyx mori* (Lepidoptera), *Apis cerana* (Hymenoptera), *Nicrophorus vespilloides* (Coleoptera), *Drosophila melanogaster* (Diptera), and *Anabrus simplex* (Orthoptera). The blue, green, yellow and orange boxes indicate the RNA recognition motif (RRM), the hinge, the Pro-rich segment, and the C-terminal domain (CTD), respectively. (**C**) A phylogenetic tree was constructed using the maximum likelihood method in MEGA6, based on *Nl*CstF64 and 17 orthologs from six different insect orders.

Secondly, *Nl*CstF64 contains a conserved RNA-recognition motif (RRM) at its N-terminus, followed by a hinge region and a CTD (Figure 2A). Notably, *Nl*CstF64 includes an additional proline-rich (Pro-rich) segment between the hinge region and the CTD. Alignment of *Nl*CstF64 with amino acid sequences from different insects showed highly conserved RRM, hinge, and CTD regions, with an abundance of proline residues in the Pro-rich segment (Figure 2B). Phylogenetic tree analysis using the maximum likelihood method clustered *Nl*CstF64 according to insect orders, confirming its conserved nature (Figure 2C).

**Figure 3:**
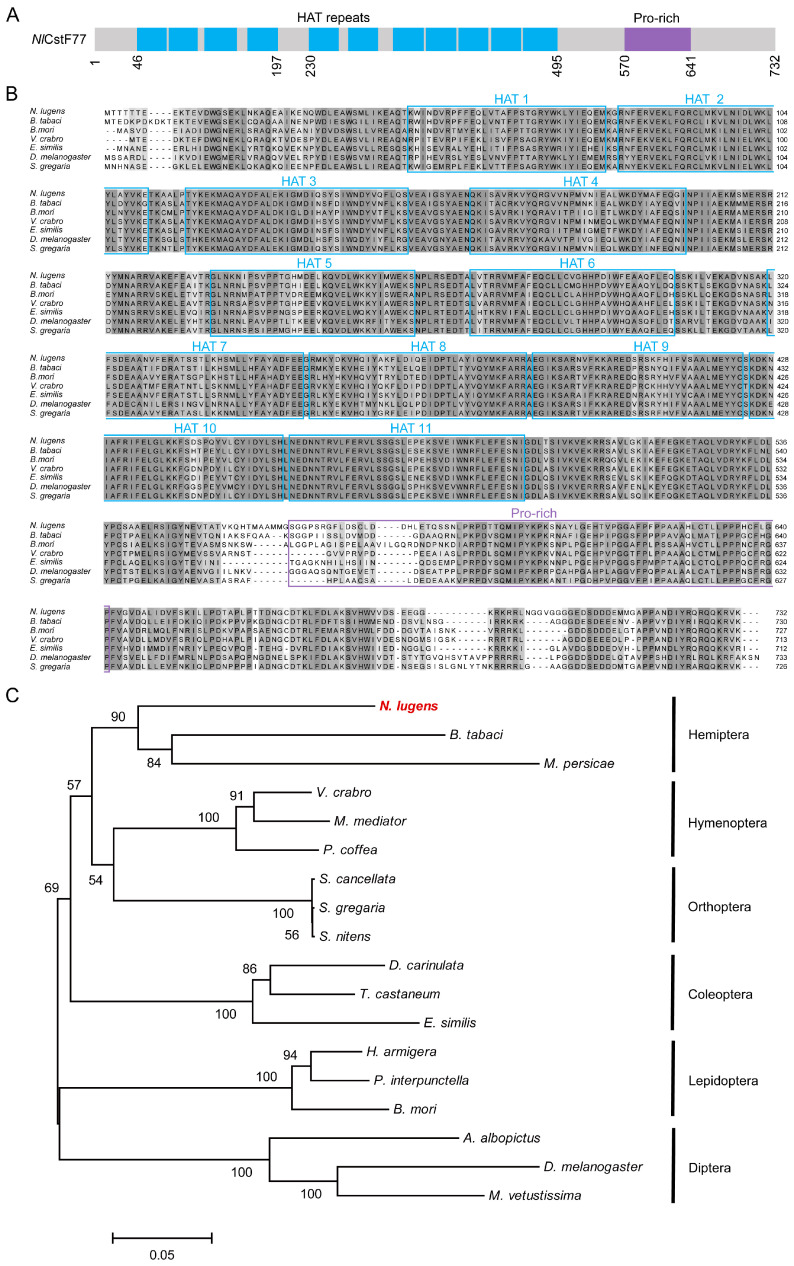
Sequence and phylogenetic analysis of *Nl*CstF77. (**A**) Schematic representation of the domain organization in the *Nl*CstF77 protein. (**B**) Alignment of amino acid sequences across six insect orders: *Bemisia tabaci* (Hemiptera), *Bombyx mori* (Lepidoptera), *Vespa crabro* (Hymenoptera), *Euwallacea similis* (Coleoptera), *Drosophila melanogaster* (Diptera), and *Schistocerca gregaria* (Orthoptera). The blue and purple boxes indicate the half-a-TPR motifs (HAT) repeat domain and the Pro-rich segment, respectively. (**C**) A phylogenetic tree was constructed using the maximum likelihood method in MEGA6, based on *Nl*CstF77 and 17 orthologs from six different insect orders.

Finally, *Nl*CstF77 features eleven half-a-TPR (HAT) repeats at its C-terminus, followed by a Pro-rich segment that binds the hinge region of CstF64 and the WD40 repeats of CstF50 (Figure 3A). Alignment of *Nl*CstF77 with sequences from various insects revealed highly conserved HAT and Pro-rich domains (Figure 3B). Phylogenetic tree analysis confirmed that *Nl*CstF77 is a conserved gene across insect orders (Figure 3C).

Overall, these analyses demonstrate that the three *NlCstF* genes are relatively conserved among insects, suggesting their potential roles in fundamental biological processes.

### 3.2. Spatiotemporal Expression Patterns of Three NlCstF Genes

**Figure 4:**
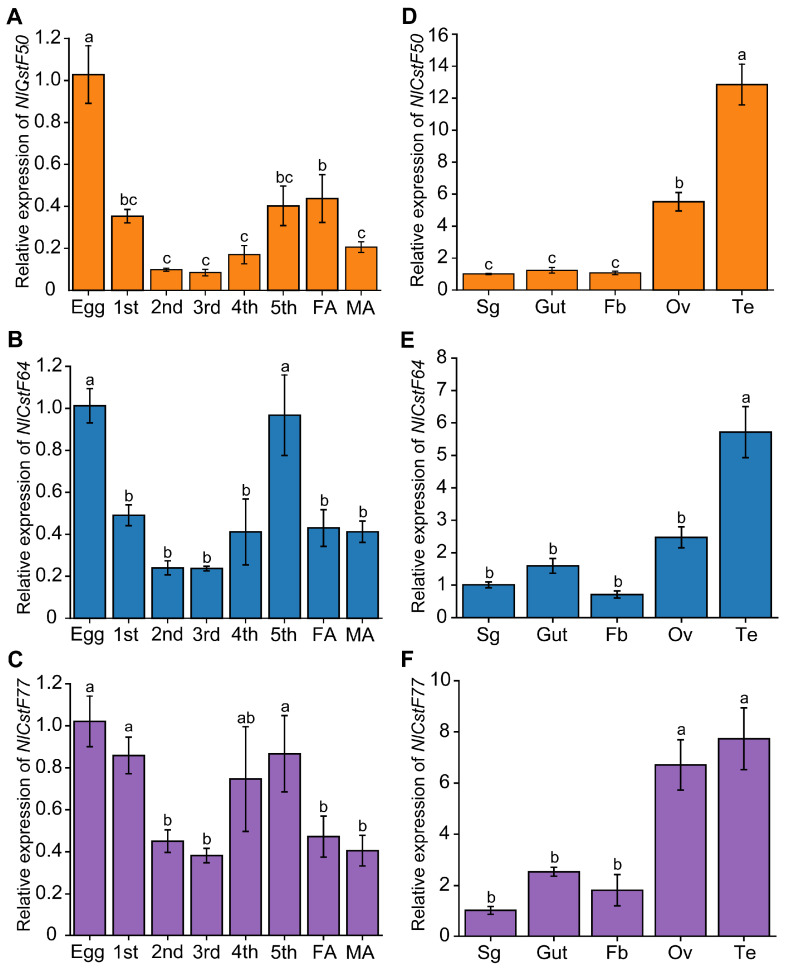
Spatiotemporal expression patterns of three *NlCstF* genes. Relative expression levels of *NlCstF50* (**A**), *NlCstF64* (**B**), and *NlCstF77* (**C**) throughout different developmental stages, including eggs, nymphs (1st to 5th instar), and adults (female adults, FA, and male adults, MA). Relative expression levels of *NlCstF50* (**D**), *NlCstF64* (**E**), and *NlCstF77* (**F**) in different tissues of *Nilaparvata lugens*. Tissues examined include the salivary glands (Sg), gut (Gut), fat body (Fb), ovaries (Ov) and testes (Te) from adults 72 h post-emergence. Data are presented as mean ± standard error of the mean (SEM) (n = 3–4), and relative gene expression was calculated using the 2^−ΔΔCT^ method. Statistical analysis was performed using one-way ANOVA followed by LSD’s post hoc test. Different letters on the bars indicate significant differences (*p* < 0.05).

To further elucidate the functional roles of the *NlCstF* genes in BPH, we examined their transcript levels across various developmental stages and tissues using quantitative real-time PCR (qRT-PCR). The results demonstrated that *NlCstF50*, *NlCstF64*, and *NlCstF77* are expressed throughout the life cycle of BPH, including in eggs, nymphs, and adults (Figure 4). Notably, the relative expression levels of all three genes were highest during the egg stage. *NlCstF50* showed elevated expression in eggs compared to other developmental stages (Figure 4A). *NlCstF64* exhibited significantly higher expression levels in eggs and 5th-instar nymphs, with reduced expression in the 1st- to 4th-instar stages and adults (Figure 4B). Similarly, *NlCstF77* was expressed at significantly higher levels in eggs, 1st-, and 5th-instar nymphs compared to the 2nd- and 3rd-instar stages and adults (Figure 4C).

In terms of tissue-specific expression, *NlCstF50*, *NlCstF64*, and *NlCstF77* were most abundantly expressed in the ovaries and testes. They also showed comparable expression levels in the salivary glands, fat body, and gut (Figure 4D–F). Overall, the consistent spatial and temporal expression patterns of *NlCstF50*, *NlCstF64*, and *NlCstF77* across different tissues and developmental stages suggest that these genes may play similar roles in the growth and development of *N. lugens*.

### 3.3. RNAi of NlCstFs Impacted Nymphal Survival

**Figure 5:**
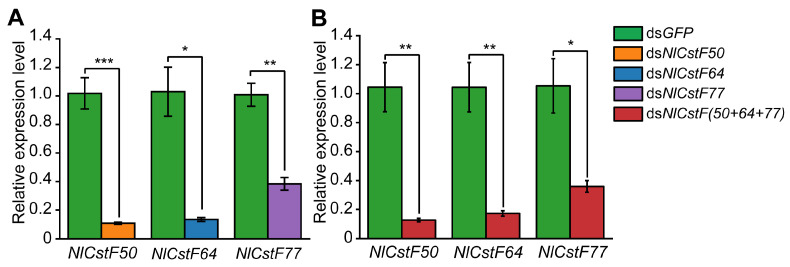
Relative expression levels of the three target *NlCstF* genes 72 h post-dsRNA injection. (**A**) Relative expression levels of *NlCstF50, NlCstF64* or *NlCstF77* were measured three days post-injection of ds*NlCstF50*, ds*NlCstF64* or ds*NlCstF77* into 3rd-instar nymphs, respectively. (**B**) Relative expression levels of *NlCstF50, NlCstF64* and *NlCstF77* were assessed three days after co-injection of ds*NlCstF(50+64+77)* (a mixture of ds*NlCstF50*, ds*NlCstF64*, and ds*NlCstF77*) into 3rd-instar nymphs. ds*GFP* was used as a control, and relative expression levels of genes were normalized using *Nl18S* as the reference gene. Data are presented as mean ± standard error of the mean (SEM) from three independent experiments. Asterisks indicate significant differences determined by a two-sided Student’s *t*-test (* *p* < 0.05; ** *p* < 0.01; *** *p* < 0.001).

To understand the function of *NlCstF* genes, dsRNA targeting individual (ds*NlCstF50*, ds*NlCstF64*, and ds*NlCstF77*) or all three (ds*NlCstF(50+64+77)*) genes were injected into 3rd-instar nymphs of BPH. The expression of the targeted genes was measured on day 3 post-injection using qRT-PCR. The results indicated that the expression levels of *NlCstF50*, *NlCstF64*, and *NlCstF77* were significantly reduced in the ds*NlCstF50*, ds*NlCstF64*, and ds*NlCstF77* groups, respectively. Additionally, in the ds*NlCstF(50+64+77)* treatment group, the expression levels of all three genes decreased (Figure 5). Specifically, the expression levels of *NlCstF50, NlCstF64,* and *NlCstF77* in the single-gene dsRNA groups decreased to 10%, 13%, and 38% of the ds*GFP* control group, respectively (Figure 5A). Similarly, in the ds*NlCstF(50+64+77)* combined injection group, the expression levels of these three genes decreased to 13%, 17%, and 36% of the ds*GFP* control group, respectively (Figure 5B). These results indicate that RNAi effectively knocked down the expression of the target genes.

**Figure 6:**
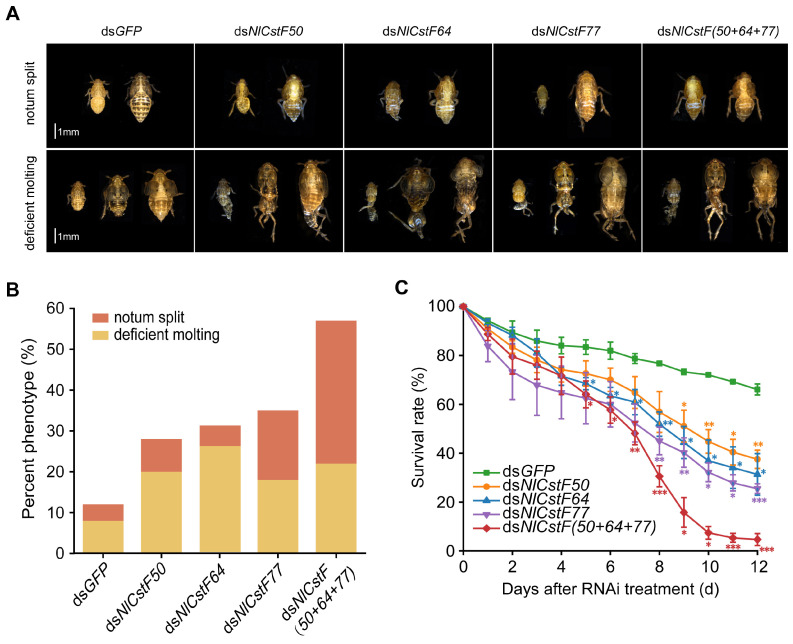
Effects of RNAi on lethal phenotypes and survival rate in *N. lugens*. Third-instar nymphs were treated with dsRNA targeting *NlCstF* genes, with ds*GFP* serving as a control. (**A**) Lethal phenotypes observed following dsRNA injection were categorized into two classes: notum split and deficient molting. Scale bar = 1 mm. (**B**) Percentage of lethal phenotypes following dsRNA injection (n > 100). (**C**) Survival rates following injection with ds*NlCstF50*, ds*NlCstF64*, ds*NlCstF77* and ds*NlCstF(50+64+77)* (a mixture of ds*NlCstF50*, ds*NlCstF64*, and ds*NlCstF77*). Each treatment was based on three biological replicates. Data are presented as mean ± standard error of the mean (SEM). Asterisks indicate significant differences determined by a two-sided Student’s *t*-test (* *p* < 0.05; ** *p* < 0.01; *** *p* < 0.001).

Insects injected with dsRNAs targeting either individual genes or all three *NlCstF* genes simultaneously exhibited similar lethal phenotypes (Figure 6). These phenotypes primarily fell into two categories (Figure 6A). The first category involved the old cuticles splitting open at the notum, exposing the underlying notum (notum split). The second category was characterized by the old cuticle remaining attached to the BPH’s abdomen and hind legs, resulting in unsuccessful shedding during eclosion (deficient molting). The proportions corresponding to each phenotype were also recorded (Figure 6B). For the first phenotype, nymphs injected with ds*NlCstF(50+64+77)* exhibited a higher rate (35%) compared to those injected with dsRNAs targeting individual genes, such as ds*NlCstF77* (17%). For the second phenotype, nymphs injected with ds*NlCstF50*, ds*NlCstF64,* ds*NlCstF77*, and ds*NlCstF(50+64+77)* displayed similar rates of 20%, 26%, 18%, and 22%, respectively, all of which were significantly higher than those observed with ds*GFP* injection (8%).

Finally, the survival rate trends among nymphs injected with ds*NlCstF50*, ds*NlCstF64*, and ds*NlCstF77* were similar but differed markedly from those injected with *NlCstF(50+64+77)* (Figure 6C). At 12 days post-injection, the survival rates for nymphs injected with ds*NlCstF50*, ds*NlCstF64*, and ds*NlCstF77* were 37.5%, 31.3%, and 25.5%, respectively, whereas the survival rate for nymphs injected with *NlCstF(50+64+77)* was significantly lower at 4.6%. Overall, these results indicate that knocking down either individual genes or all three *NlCstF* genes simultaneously has a significant impact on nymphal survival, disrupting their molting process.

### 3.4. Effects of NlCstFs on the Reproduction of N. lugens

**Figure 7:**
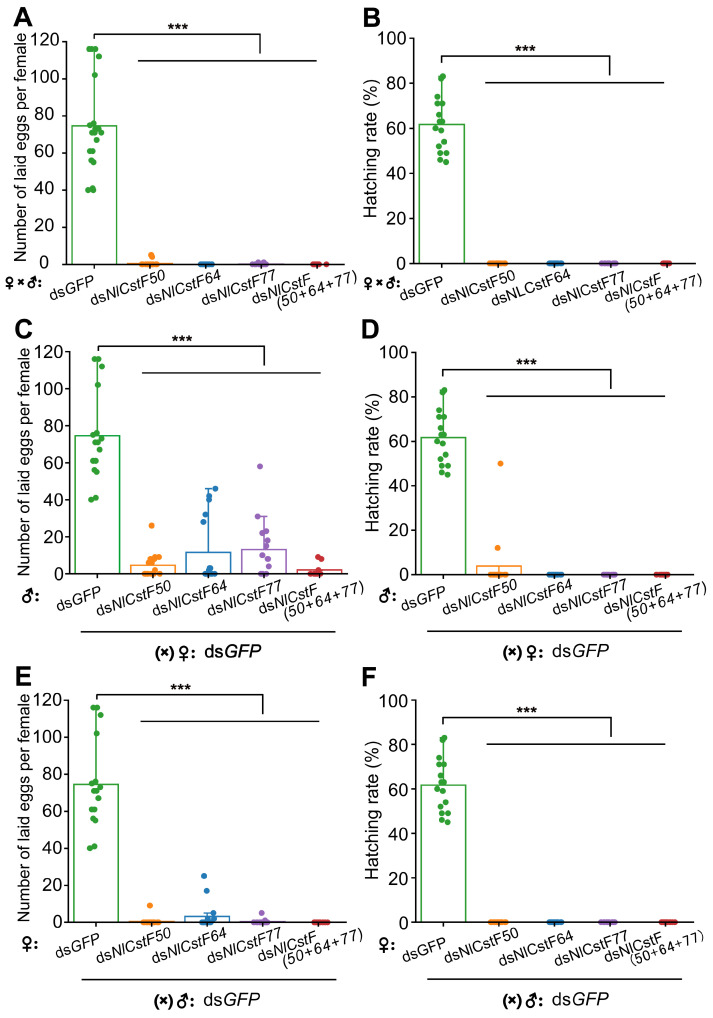
Effects of RNAi on egg-laying and hatchability in *N. lugens.* Fifth-instar nymphs were treated with dsRNA targeting *NlCstF* genes, with ds*GFP* serving as a control. Three days post-adult emergence, females were allowed to mate with males, and egg deposition was observed over a three-day period. (**A**) Number of eggs laid per female injected with dsRNA, mated with males injected with either ds*GFP*, ds*NlCstF50*, ds*NlCstF64*, ds*NlCstF77*, or ds*NlCstF(50+64+77)*. (**B**) Hatching rate of eggs from each mating pair described in (**A**). (**C**) Number of eggs laid per female injected with ds*GFP*, mated with males injected with either ds*GFP*, ds*NlCstF50*, ds*NlCstF64*, ds*NlCstF77* or ds*NlCstF(50+64+77)*. (**D**) Hatching rate of eggs from each mating pair described in (**C**). (**E**) Number of eggs laid per female injected with dsRNA, mated with males injected with ds*GFP*. (**F**) Hatching rate of eggs from each mating pair described in (**E**). Data are presented as mean ± standard error of the mean (SEM). Asterisks indicate significant differences determined by a two-sided Student’s *t*-test (*** *p* < 0.001).

The three *NlCstF* genes were found to be highly expressed in reproductive tissues, particularly in the testes, suggesting a potential role in BPH reproduction. However, injecting dsRNAs for *NlCstF* genes into 3rd-instar nymphs resulted in severe lethality, yielding an insufficient number of adults for reproduction experiments. Consequently, 5th-instar nymphs were used for injection with four different dsRNAs targeting the *NlCstF* genes. Males and females injected with ds*GFP*, ds*NlCstF50*, ds*NlCstF64*, ds*NlCstF77*, or ds*NlCstF(50+64+77)* were allowed to mate with each other. Subsequently, the number of unhatched eggs and newly hatched nymphs was recorded and analyzed. Females injected with ds*NlCstF50*, ds*NlCstF64*, ds*NlCstF77*, or ds*NlCstF(50+64+77)* virtually did not deposit any eggs, in contrast to the ds*GFP* control females, which laid an average of 75 eggs (Figure 7A). Likewise, none of the eggs from the dsRNA treatments targeting *NlCstF* genes hatched into nymphs, whereas 61.7% of the eggs from the ds*GFP* treatment developed into nymphs (Figure 7B).

To determine the roles of males and females in reproduction after silencing the *NlCstF* genes, females from the ds*GFP* treatment were separately mated with males from the dsRNA treatments targeting *NlCstF* genes (Figure 7C,D), and vice versa (Figure 7E,F). In the former condition (Figure 7C,D), females that mated with males injected with ds*NlCstF50,* ds*NlCstF64*, ds*NlCstF77*, and ds*NlCstF(50+64+77)* laid an average of 5, 12, 13, and 2 eggs, respectively, which were significantly fewer than the ds*GFP* control (Figure 7C). Except for the eggs from mating with males injected with ds*NlCstF50*, which had a hatching rate of 3.9%, none of the other eggs from mating with males injected with ds*NlCstF64*, ds*NlCstF77*, and ds*NlCstF(50+64+77)* developed into nymphs (Figure 7D). In the latter condition (Figure 7E,F), only females injected with ds*NlCstF6*4 laid an average of 3 eggs, whereas females injected with ds*NlCstF50*, ds*NlCstF77*, and ds*NlCstF(50+64+77)* did not deposit any eggs (Figure 7E). None of the eggs from the dsRNA treatments targeting *NlCstF* genes hatched into nymphs (Figure 7F). These results indicate that the three *NlCstF* genes play crucial roles in reproduction in both sexes.

**Figure 8:**
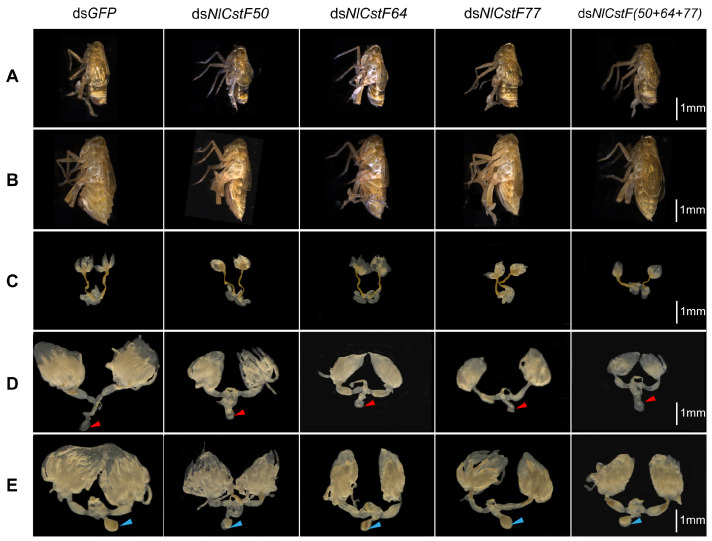
Effects of RNAi on the morphology and the internal reproductive organs in *N. lugens.* Morphological observation of males (**A**) and females (**B**) were conducted 3 days post-adult eclosion. Testes (**C**) and ovaries (**D**) were dissected from dsRNA-injected males and females, respectively, 3 days post-emergence. (**E**) Ovaries were dissected from dsRNA-injected females 6 days post-emergence. Red triangles indicate bursa copulatrix without spermatophores (unfertilized), and blue triangles indicate bursa copulatrix with spermatophores (unfertilized). Fifth-instar nymphs were treated with dsRNA targeting *NlCstF* genes, with ds*GFP* serving as a control. Scale bar = 1 mm (**A**–**E**).

Next, the morphology of males and females and the development of testes and ovaries were examined after injection with four different dsRNAs targeting the *NlCstF* genes. No morphological differences were observed between males with silenced *NlCstF* genes and the ds*GFP* control males (Figure 8A). However, the abdomens of females injected with ds*NlCstF50*, ds*NlCstF64,* ds*NlCstF77*, and ds*NlCstF(50+64+77)* were obviously smaller than those of dsGFP control females 3 days post-adult emergence (Figure 8B). The three spermathecal ducts of testes dissected from males injected with ds*NlCstF50*, ds*NlCstF64*, ds*NlCstF77*, and ds*NlCstF(50+64+77)* were abnormal compared to those of ds*GFP* control males 3 days post-adult emergence (Figure 8C). The ovaries dissected from females injected with ds*NlCstF50*, ds*NlCstF64*, ds*NlCstF77*, and ds*NlCstF(50+64+77)* showed delayed development and were smaller than those of ds*GFP* control females 3 and 6 days post-adult emergence (Figure 8D,E). Finally, seminal vesicles were found in the ovaries of females 6 days post-adult emergence (Figure 8E), suggesting that successful insemination occurred in males mating with females in all experiments. Overall, these results indicate that knocking down the three *NlCstF* genes results in defective testis and ovarian development, thereby impacting the reproduction of BPH.

## 4. Discussion

The CstF complex is critical for mRNA 3′ end processing, playing a significant role in pre-mRNA maturation and transcription regulation [8]. This study investigates the functions of CstF complex genes (*NlCstF50*, *NlCstF64*, and *NlCstF77*) in the Hemiptera pest, *N*. *lugens*. Sequence analysis of *Nl*CstF50, *Nl*CstF64, and *Nl*CstF77 from *N*. *lugens* revealed that these genes are conserved across various insect orders (Figure 1, Figure 2 and Figure 3). This conservation suggests they likely share similar functional domains and roles in RNA processing and gene regulation, such as the WD40 domain in CstF50, the RRM domain in CstF64, and the HAT domain in CstF77. Notably, the domain repeats in some CstF proteins of *N*. *lugens* differ from those in mammalian CstFs. For instance, the WD40 domain repeats are critical for interaction with CstF77 [10,11]. The *Nl*CstF50 protein contains six repeats, while the human CstF50 protein has seven, with the sixth repeat absent in *Nl*CstF50. Deletion of the seventh repeat reduces binding affinity [11], suggesting that the sixth repeat may not significantly impact the interaction between *Nl*CstF50 and *Nl*CstF77. Similarly, the HAT domain repeats are essential for CstF77 dimerization. The *Nl*CstF77 protein has eleven repeats, whereas the human CstF77 protein has twelve, divided into HAT-N (first five repeats) and HAT-C (sixth to twelfth repeats) [17,18,19]. The HAT-C domains are crucial for dimeric association [33]. The missing HAT repeat in *NlCstF77*, located within the HAT-N region, suggests that HAT-C domains are involved in dimerization across organisms. Thus, sequence analysis of the CstF complex in *N*. *lugens* indicates conservation with other eukaryotic sequences and a similar role in mRNA 3′ end processing.

Mutations in CstF complex components, particularly CstF77, are associated with lethal phenotypes in *Drosophila* [24], yeast [26], and *Arabidopsis* [27,28]. To explore the functions of the CstF complex genes in *N*. *lugens*, RNAi was used to inhibit the expression of one or all three genes. Knockdown of any CstF complex gene resulted in a lethal phenotype in the instar nymphs of *N*. *lugens*. The severity of the phenotype varied, reflecting the relative importance of each gene within the complex. Among single-gene knockdowns, ds*NlCstF77* treatment caused the highest mortality, while ds*NlCstF50* led to the lowest. This suggests that CstF77 is crucial for CstF complex assembly, whereas *CstF50*’s ortholog is absent in yeast. When all three genes were knocked down simultaneously, only 4.6% of BPH nymphs survived by day 12 post-injection with ds*NlCstF(50+64+77)*, indicating that the CstF complex is likely essential for nymph development.

The observed lethal phenotype in *N*. *lugens* treated with dsRNA targeting CstF genes is primarily linked to disruptions in the molting process. This effect is associated with the CstF complex’s role in pre-mRNA maturation and transcription. Impairment of this complex may affect the transcription of molting-related genes such as chitinases, chitin deacetylases, and chitin synthases, which are essential for molting in *N*. *lugens*. Specifically, knockdown of five chitinase genes (*NlCht1*, *NlCht5*, *NlCht7*, *NlCht9*, and *NlCht10*) [34], which degrade chitin in the old cuticle during molting, along with one β-N acetylhexosaminidase (*NlHex4*) [35] and three chitin deacetylases (*NlCDA1*, *NlCDA2*, and *NlCDA4*) [36], resulted in a lethal-molting phenotype similar to the notum split observed in this study (Figure 6A). This suggests that the CstF complex in *N*. *lugens* may influence the mRNA maturation of these genes or others involved in molting.

The tissue-specific expression of *NlCstF50, NlCstF64*, and *NlCstF77* indicates high levels of expression in reproductive tissues such as the ovary and testis. Suppression of one or all three genes in the CstF complex leads to impaired fertility in both sexes (Figure 7). Similar expression patterns have been observed in *Drosophila* and mammals [37,38,39]. In *Drosophila*, the Su(f) gene is predominantly expressed in the germarium during oogenesis [40], and mutations in Su(f) result in female sterility [24]. In mice, the *CstF64* gene is highly expressed in the testis, particularly in male germ cells [38,39]. Male mice deficient in *CstF64* exhibit disrupted spermatogenesis and infertility, while female fertility remains unaffected [39]. Thus, the CstF complex is crucial for reproduction across various species by influencing the mRNA polyadenylation of essential genes.

## 5. Conclusions

This study successfully identified and analyzed three *NlCstF* genes in *N. lugens.* These genes were expressed across various developmental stages and tissues, with particularly high levels observed in eggs and testes. RNAi experiments demonstrated that silencing these genes significantly reduced survival rates, the number of eggs laid per female, and hatch rates, underscoring their crucial role in the development and reproduction of *N*. *lugens*. Simultaneous silencing of all three *NlCstF* genes resulted in even more severe lethal and fertility-defective phenotypes. This suggests that targeting all three *NlCstF* genes concurrently could be an effective strategy for RNAi-based pest control, potentially mitigating the impact of BPH on rice crops.

## Data Availability

The data presented in this study are available in the article and its Supplementary Materials.

## Supplementary Materials

The following supporting information can be downloaded at: https://www.mdpi.com/article/10.3390/insects15110867/s1, Table S1: List of primers used in this study; Table S2: List of orthologs of three CstF proteins in the phylogenetic analysis; Figure S1: Nucleotide sequence alignment and dsRNA localization of the cloned sequence of the *NlCstF50*, *NlCstF64,* and *NlCstF77* genes are presented. The dsRNA regions for *NlCstF50*, *NlCstF64,* and *NlCstF77* are highlighted with orange, blue, and purple boxes, respectively.
