# Supplementary material for: Identification and Functional Analysis of Three NlCstF Genes in Nilaparvata lugens
Source: Insects. 2024 Nov 5;15(11):867. doi: 10.3390/insects15110867 (PMC11595156; doi:10.3390/insects15110867)
Supplement: Supplementary file 1 [file insects-15-00867-s001.zip › Table S1 CstF primer-revision.pdf]

Table S1: List of primers used in this study

Name of primers	Primers sequences (5′ → 3′)	Sizes (bp)
	**For cDNA cloning**	
NlCstF50-1F	ATGAAGGAAACGGAAAATCCCCAGG	
NlCstF50-1R	TCAGTGTACAAGTCGCTTGTACCAG	
NlCstF50-2F	TGGTCTTGATTTGGAGTTCGAGACG	
NlCstF50-2R	AATTTATAAAAAAACACGATTTATT	
NlCstF64-1F	ACTAATAGCCAAGTTTCCAT	
NlCstF64-1R	ACTGGGTTTGTCTTGTGC	
NlCstF64-2F	GCACAAGACAAACCCAGT	
NlCstF64-2R	TAATTGATCTTGAGGAGCAAAAGCG	
NlCstF64-3F	GGTGCAATGAATCAAGACATGGACA	
NlCstF64-3R	CAGCTGAAAGAAGGATTAGTTTTT	
NlCstF77-1F	CAAACTTGATCATCAACAAAGCAGG	
NlCstF77-1R	CGTTCAAATGTGATAAGTAGTCGAT	
NlCstF77-2F	AGTTATTTCAACGATGCCTCATGAA	
NlCstF77-2R	CGGTATCAGGCAAGAGTATCTTGCT	
NlCstF77-3F	ACGACCACCTGGAGACAC	
NlCstF77-3R	TGACCCTTCAGCCACTTA	
	**For qPCR**	
Q*NlCstF50*-F	GGAATACGACGGGCAGAC	
Q*NlCstF50*-R	TTGAGTAGACCGAGGGAGTG	
Q*NlCstF64*-F	GCAGTTGGCATACGCATTG	
Q*NlCstF64*-R	GGCGGGTTGTTACCTTCG	
Q*NlCstF77*-F	CGACATCTACAGGCAGCGACAG	
Q*NlCstF77*-R	GACCCTTCAGCCACTTATTCT	
Q*Nl18S*-F	CGCTACTACCGATTGAA	
Q*Nl18S*-R	GGAAACCTTGTTACGACTT	
	**For dsRNA synthesis**	
ds*NlCst50*-F	TAATACGACTCACTATAGGGGAAAATCCCCAGGACATC	**386**
ds*NlCst50*-R	TAATACGACTCACTATAGGGCTGCCCATCCATGCTGAA	
ds*NlCstF64*-F	TAATACGACTCACTATAGGGTGAGATTGGCGGTCGTAT	**348**
ds*NlCstF64*-R	TAATACGACTCACTATAGGGACTGGGTTTGTCTTGTGC	
ds*NlCstF77*-F	TAATACGACTCACTATAGGGGCCCAAATCCAATGCCTAC	**325**
ds*NlCstF77*-R	TAATACGACTCACTATAGGGCGCTCCCATCATCTCGTC	
ds*GFP*-F	TAATACGACTCACTATAGGGAGAATGAGTAAAGGAG AAGAACTTTTC	**495**
ds*GFP*-R	TAATACGACTCACTATAGGGAGATTTGTATAGTTCAT CCATGCCATGT	

Sizes: sizes (in bp) of the three dsRNA molecules produced to target the respective transcripts.
