# Supplementary material for: Identification and Functional Analysis of Three NlCstF Genes in Nilaparvata lugens
Source: Insects. 2024 Nov 5;15(11):867. doi: 10.3390/insects15110867 (PMC11595156; doi:10.3390/insects15110867)
Supplement: Supplementary file 1 [file insects-15-00867-s001.zip › Table S2 CstF proteins-revision.pdf]

Table S2: List of orthologs of three CstF proteins in the phylogenetic analysis

Gene name	number	Species	GenBank accession number
	1	*Nilaparvata lugens*	PQ226090
	2	*Laodelphax striatellus*	RZF39249.1
	3	*Lygus hesperus*	JAQ11147.1
	4	*Hylaeus anthracinus*	XP_054002028.1
	5	*Venturia canescens*	XP_043280536.1
	6	*Phymastichus coffea*	XP_058798990.1
	7	*Schistocerca cancellata*	XP_049788191.1
	8	*Schistocerca gregaria*	XP_049832347.1
	9	*Schistocerca nitens*	XP_049815072.1
CstF50	10	*Diorhabda sublineata*	XP_056635379.1
	11	*Zophobas morio*	XP_063917885.1
	12	*Euwallacea similis*	XP_066260429.1
	13	*Achroia grisella*	XP_059056600.1
	14	*Galleria mellonella*	XP_052757234.1
	15	*Bombyx mori*	XP_004926336.1
	16	*Culicoides brevitarsis*	XP_063696576.1
	17	*Eupeodes corollae*	XP_055902523.1
	18	*Drosophila melanogaste*	NP_651883.1
	1	*Apis cerana*	XP_016922497.1
	2	*Melipona quadrifasciata*	KOX71803.1
	3	*Diachasma alloeum*	XP_015127609.1
	4	*Anabrus simplex*	XP_066994118.1
	5	*Schistocerca americana*	XP_046990195.1
	6	*Schistocerca nitens*	XP_046990195.1
	7	*Nilaparvata lugens*	PQ226091
	8	*Bemisia tabaci*	XP_018900972.1
	9	*Myzus persicae*	XP_022168450.1
CstF64	10	*Acanthoscelides obtectus*	CAK1630193.1
	11	*Dalotia coriaria*	XP_065171041.1
	12	*Nicrophorus vespilloides*	XP_017769458.1
	13	*Bombyx mori*	XP_004930566.1
	14	*Helicoverpa armigera*	XP_063893824.1
	15	*Spodoptera litura*	XP_022826242.1
	16	*Anopheles moucheti*	XP_052889069.1
	17	*Culicoides brevitarsis*	XP_063704380.1
	18	*Drosophila melanogaster*	NP_477453.1

	1	*Nilaparvata lugens*	PQ226092
	2	*Bemisia tabaci*	XP_018909511.1
	3	*Myzus persicae*	XP_022173547.1
	4	*Vespa crabro*	XP_046832196.1
	5	*Microplitis mediator*	XP_057319442.1
	6	*Phymastichus coffea*	XP_058803933.1
	7	*Schistocerca cancellata*	XP_049767009.1
	8	*Schistocerca gregaria*	XP_049839638.1
CstF77	9	*Schistocerca nitens*	XP_049793735.1
	10	*Diorhabda carinulata*	XP_057664078.1
	11	*Tribolium castaneum*	XP_015836548.1
	12	*Euwallacea similis*	XP_066255675.1
	13	*Helicoverpa armigera*	XP_063898730.1
	14	*Plodia interpunctella*	XP_053622101.1
	15	*Bombyx mori*	XP_004925975.1
	16	*Aedes albopictus*	XP_019553197.1
	17	*Drosophila melanogaster*	NP_001104480.2
	18	*Musca vetustissima*	XP_061394293.1
